# Impact of Prebiotic β-glucan Treatment at Juvenile Age on the Gut Microbiota Composition and the Eventual Type 1 Diabetes Onset in Non-obese Diabetic Mice

**DOI:** 10.3389/fnut.2021.769341

**Published:** 2021-11-03

**Authors:** Harrison B. Taylor, Chenthamarakshan Vasu

**Affiliations:** Department of Microbiology and Immunology, College of Medicine, Medical University of South Carolina, Charleston, SC, United States

**Keywords:** yeast β-glucan, autoimmunity, type 1 diabetes, gut microbiota, immune modulation β-glucan, gut mucosa, immune regulation, microbiota

## Abstract

Complex dietary polysaccharides such as β-glucans are widely used for their anti-inflammatory properties. We reported before that oral administration of Yeast β-glucan (YBG) in adult mice can help delay type 1 diabetes (T1D) onset and suppress gut inflammation through modulation of the structure and function of gut microbiota. Since juvenile age is characterized by profoundly changing immature gut microbiota, we examined the impact of oral treatment with YBG in non-obese diabetic (NOD) mice at this age. Juvenile mice that received daily oral administration of YBG starting at 15 days of age for 7 or 30 days were examined for changes in gut microbiota, immune characteristics, and T1D incidence. Mice that received YBG for 30 days but not 7 days, showed considerable changes in the composition and diversity of fecal microbiota as compared to controls. Predictive functional analysis, based on 16S rDNA sequences, revealed overrepresentation of glycan biosynthesis and metabolism, energy metabolism, and fatty acid biosynthesis pathways in mice that received YBG for 30 days. Immune phenotype of the colon showed skewing toward immune regulatory and Th17 cytokines with increases in IL-10, IL-17, and IL-21 and a decrease in TNF-α, although increases in some pro-inflammatory cytokines (IL-1b, IFN-γ) were observed. Most importantly, mice that received YBG treatment for 30 days showed significantly suppressed insulitis and delayed onset of hyperglycemia compared to controls. Overall, this study suggests that oral consumption of YBG beginning at pre-diabetic juvenile ages could have positive maturational changes to gut microbiota and immune functions and could result in a delay in the disease onset in those who are pre-disposed to T1D.

## Introduction

The use of β-glucans, complex dietary polysaccharides (CDP) with prebiotic properties, as dietary supplements could confer a multitude of benefits to host digestive, cardiovascular, and immune systems (1–5). Typically comprised of a β-1,3-D-glucan backbone with variability in the presence and pattern of β-1,4- and β-1,6-linked side chains and branching (6), these molecules are derived from many natural sources such as yeast (6), mushrooms (7), algae (8), and cereals (9). The impact of different types of β-glucan on gut inflammation and autoimmune disease outcomes appears to be dependent in part on its source, size, solubility, and method of administration (1, 10–13). Our previous reports (4, 5) indicate that oral treatment of murine models of type 1 diabetes (T1D) and colitis with highly purified β-glucans, starting at pre-disease/clinical stage, has an ameliorating effect on the disease outcomes. These beneficial effects of β-glucan appear to be mediated in part through changes in the gut microbiota composition and function.

T1D is an autoimmune disease that results from immune attack on the pancreatic β cells responsible for producing insulin and is characterized by failure of the pancreas to produce sufficient levels of insulin (14, 15). In human and rodent models, the gut microbiota at the onset T1D appear to be characterized by higher proportions of the Bacteroidetes phylum, primarily *Bacteroides*, and a diminished capacity for short-chain fatty acid (SCFA) biosynthesis (16–18). On the other hand, our reports have shown that oral administration of β-glucans significantly altered the gut microbiota through reduction of the phylum Firmicutes and enrichment of communities belonging to phylum Verrucomicrobia and Bacteroidetes (4, 5, 19) and ameliorated disease outcomes in T1D and colitis. We showed that oral administration of Yeast-derived β-glucan (YBG) and algal-derived β-glucan (paramylon, PM) caused overrepresentation of gut microbiota functions related to carbohydrate metabolism, including glycan and simple sugar metabolisms (4, 19). Importantly, in murine models of both type 1 and 2 diabetes, such changes in gut microbiota functions were associated with improved disease outcomes and reduced inflammation (4, 20).

Although T1D onset can occur at any age, it is mostly detected at juvenile age. Juvenile age is characterized by profoundly changing immature gut microbiota, which matures and stabilizes gradually during the early life (21, 22). It is generally believed that dietary practices during these ages could influence the structure and function core microbiota at later ages (23, 24). Since our previous work (4, 5, 19) showed that treatment of adult mice with β-glucan is effective in altering the gut microbiota composition leading to improved immune regulation and disease outcomes in T1D and colitis, we examined if oral administration of β-glucan at juvenile age, which involves changing/maturing gut microbiota, impacted the composition and function of microbiota and the eventual T1D onset. Here, we show that at the juvenile stage, prolonged treatment (30 days) with YBG alters the gut microbiota, immune phenotype, and disease outcomes in NOD mice. Overall, this study shows the potential of oral consumption of dietary β-glucan beginning at younger pre-diabetic ages in delaying the onset of T1D in genetically predisposed individuals.

## Materials and Methods

### Mice and Treatments

NOD/LtJ (NOD/ShiLtJ) mice were originally purchased from the Jackson Laboratory (Bar Harbor, ME), with breeding colonies of the strain maintained in the specific pathogen-free (SPF) facility at the Medical University of South Carolina (MUSC). Female mice were used in each set of experiments. Mice were maintained on a standard autoclaved diet containing 44.2% (wt:wt) carbohydrates, 18.6% (wt:wt) protein, and 6.2% (wt:wt) fat (Teklad Global 18% Protein Rodent diet, Harlan Laboratories) and acidified (pH = 3.0–3.2) RO water as described in our previous report (5, 25) to reduce the chances of contamination between colonies within the facility. Monitoring of solid food and water intake indicated no difference in the eating habits between mice of different treatments (Supplementary Table 1). All studies were approved by MUSC's animal care and use committee.

Two-week old juvenile female NOD/LtJ were given either 100 μl 0.9% saline solution as control treatment or 100 μl (or 100 μg) of YBG solution, prepared from highly purified YBG (from Baker's yeast, *Saccharomyces cerevisiae*; >98% pure; Sigma-Aldrich) as indicated previously (26). Mice were treated every 24 h for either 7 or 30 days *via* oral gavage. All mice were weaned at 21 days of age, while treatment progressed in the case of those treated for 30 days. To ensure that the pre-treatment microbiota was comparable in control and YBG treated mice, dams were originated from a single cage and pups were pooled and randomized on day 14 prior to treatment initiation. Therefore, control and YBG treated mice were originated from the same pool of pups. Fecal pellets were collected from 7-day treated mice (*n* = 5 for control treatment and *n* = 5 for YBG treatment) and 30-day treated mice (*n* = 5 for control treatment and *n* = 5 for YBG treatment) immediately prior to treatment initiation and 24 h after final treatment for microbiota analysis. Cohorts of 7-day treated (*n* = 10 for control treatment and *n* = 10 for YBG treatment) and 30-day treated (*n* = 13 for control treatment and *n* = 13 for YBG treatment) mice were monitored to detect hyperglycemia by measuring blood glucose levels *via* the tail vein every week beginning at 8 weeks of age until 30 weeks using the Contour^®^ blood glucose test strips and an Ascensia Contour blood glucose meter (Bayer, Leverkusen, Germany). Mice with a blood glucose level >250 mg/dL for 2 consecutive weeks were considered diabetic. Cohorts of 7-day treated mice (*n* = 5 for control treatment and *n* = 5 for YBG treatment) and 30-day treated mice (*n* = 4 for control treatment and *n* = 4 for YBG treatment) were euthanized 24 h after final treatment for obtaining intestinal tissues for qPCR assay to determine the expression profiles of cytokines. Pancreatic tissues were obtained from additional cohorts of 30-day treated mice (*n* = 4 for control treatment and *n* = 4 for YBG treatment) for determining insulitis severity.

### 16S RRNA Gene Sequencing and Microbiota Analyses

DNA was isolated from fecal pellets as described previously (25), with a few modifications. Pellets were resuspended in 50 mM Tris buffer (50 mM Tris, 1 mM EDTA, and 0.2% β-mercaptoethanol, pH = 7.5). After incubation at 95°C, lysis and protein digestion were performed using 10% SDS and Proteinase K. Phase separation of the DNA was performed with a 1:1 mixture of TRIZOL and chloroform and spun down at 14,000 rpm. The supernatant was transferred and then the DNA was precipitated using 0.1 mol/L sodium acetate and isopropanol. DNA was washed twice with 70% ethanol, air dried, and then resuspended in 100 μl sterilized milliQ H_2_O. The V3-V4 region of the 16S ribosomal RNA (rRNA) gene was sequenced on an Illumina MiSeq platform by the NC State University Genomic Sciences Laboratory (Raleigh, NC, USA). Sequence processing and classification was performed using version 1.44.3 of the mothur software package (27). The GreenGenes database (version 13.5) was used as a reference for sequencing alignment and classification into operational taxonomic unites (OTUs) using a 0.03 cutoff (28). Normalization of the data was performed by rarefying to 8,000 sequences (the number of sequences in the smallest sized sample). The OTU biome file generated was used as described previously (19) for analysis using MicrobiomeAnalyst (29) to measure alpha (Chao1, Shannon index) and beta diversity and for predictive functional analysis *via* PICRUSt (30) and iVikodak (31). Sequencing data was also analyzed employing Illumina Metagenomics application (Version 1.10) for additional taxonomic classification of 16S rRNA targeted amplicon reads using a more recently curated taxonomic database (RefSeq RDP 16S v3 database), which is based on FASTA from: https://benjjneb.github.io/dada2/training.html. The raw sequence reads used for these analyses can be found under the accession number PRJNA761366 in the Sequence Read Archive on the NCBI server.

### Determination of Cytokine Expression Levels

Twenty-four hours after treatment, a subset of mice treated with saline or YBG for 30 days was euthanized *via* CO_2_ (flow rate of 20% displacement/minute) and the colons were collected and flushed with 1x PBS supplemented with Antibiotic/Antimicrobial solution. Pieces 1 cm in length were collected from the distal portion of the colon and stored at −80°C prior to RNA extraction using TRIZOL reagent (Invitrogen). cDNA synthesis was performed on 5 μg RNA per sample using Superscript first-strand cDNA kit (Invitrogen) and then diluted 1:4 with sterilized milliQ H_2_O. Cytokine-specific primer sets were used in qPCR assay to detect the expression levels of IL-1β, IL-4, IL-6, IL-9, IL-10, IL-12, IL-17, IL-21, IL-22, TNF-α, IFN-γ, and Raldh1A2 as described previously (4). Reactions of 10 μl were prepared for each sample containing 5 μl of SsoAdvanced Universal SYBR^®^ Green Supermix (Bio-Rad, California, USA), 0.5 μl 1X forward primer, 0.5 μl 1X reverse primer, and 3.5 μl H_2_O. Reaction conditions included an initial 3 min incubation at 95°C followed by 35 cycles of 95°C for 30 s and 55°C for 30s. The melting curve step was performed by increasing 0.5°C every 10 s from 65 to 95°C.

### Insulitis

The pancreata collected from the mice euthanized 24 h after treatment were fixed in 10% formaldehyde, and 5 μm sections were subjected to hematoxylin and eosin (H&E) staining. Slides of each sampled pancreata were prepared in triplicate with three sections on each slide. H&E-stained sections were analyzed for the degree of insulitis using the following grading system: 0 = no islet cell infiltration, 1 = peri-insulitis with ~5% or less islet infiltration, 2 = ~5–25% islet infiltration, 3 = ~25–50% infiltration, and 4 = >50% infiltration, as described previously (25). At least 250 islets per group (at least 50 islets/mouse) were examined and graded.

### Statistical Analyses

Statistical analysis, including calculation and comparison of means, standard deviation (SD), and statistical significance as measured *via p*-value calculation were performed on data collected for disease progression and immune phenotype analyses using GraphPad Prism v9 (San Diego, CA). A Log-rank test was performed to analyze the difference in the disease onset timing. Unpaired *t*-tests were used to analyze the significance of data collected from RT-PCR. GraphPad prism employs *F*-test to compare the variance of two groups and we considered unpaired *t*-test for determining statistical significance when two groups showed equal variances. To analyze differences in diversity within different gut microbiota between treatments, MicrobiomeAnalyst (29) was used, with α-diversity and β-diversity analyzed *via* Mann-Whitney and permutational multivariate ANOVA (PERMANOVA) methods, respectively. STAMP (32) was used to analyze differences in specific taxa between communities as well as differences in microbial function within the gut microbiota using the Benjamini and Hochberg correction method (33).

## Results

### Diversity and Community Structure Were Influenced by Prolonged YBG Treatment in Juvenile Mice

**Figure 1:**
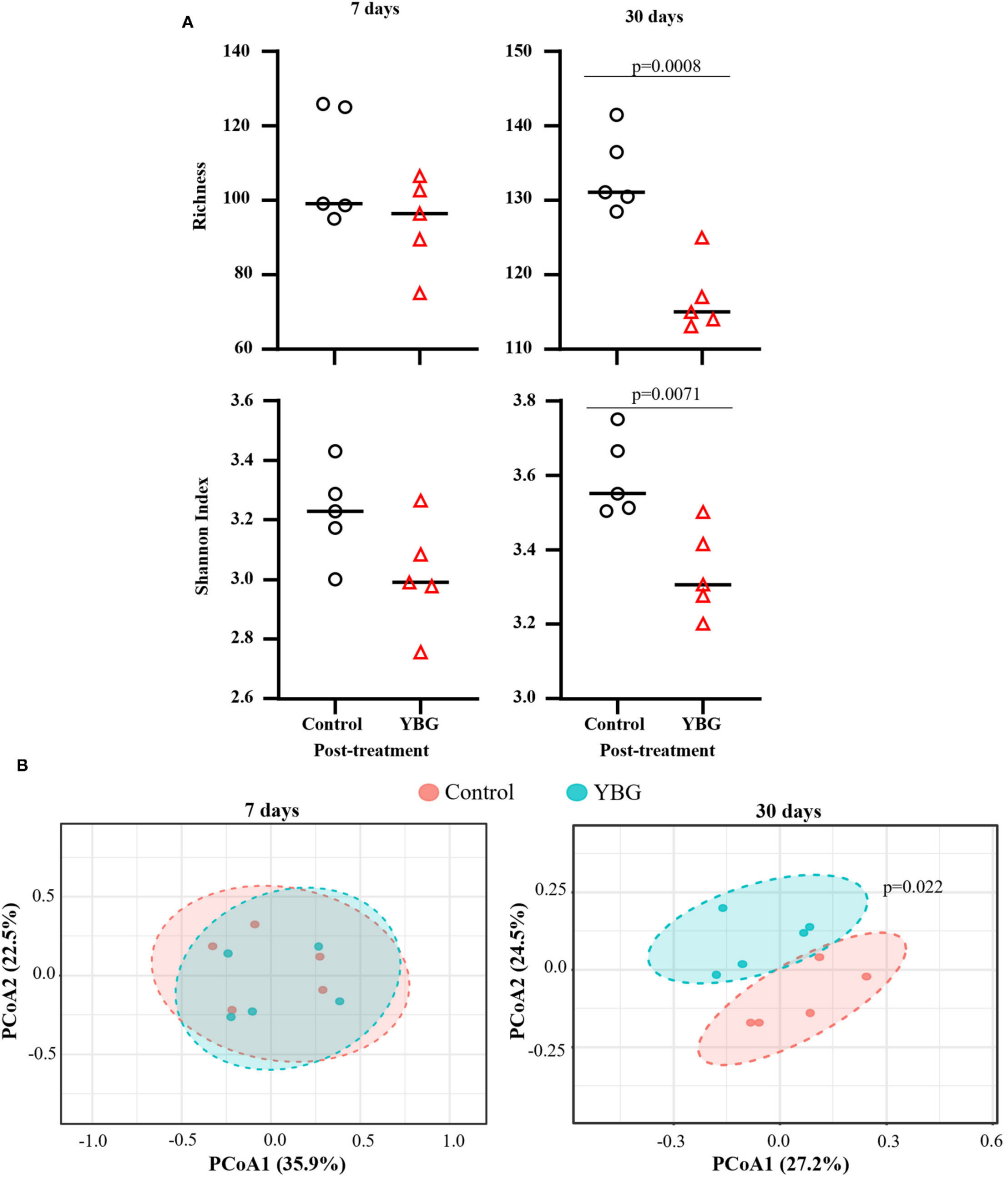
Diversity and community structure of fecal microbiota are significantly altered with 30-day YBG treatment beginning at juvenile age in NOD mice. Two-week-old female NOD mice were given 0.9% saline solution (control) or YBG suspension (100 μg/day) for 7 or 30 consecutive days *via* oral gavage. Mice for both control saline and YBG treated groups of each treatment regimen (7 or 30 days) were originated from a single pool of pups. Fecal samples collected after 7 days (5 mice/group) and 30 days (5 mice/group) of YBG treatment were subjected to 16S rRNA gene sequencing and analyzed as described in the section Materials and Methods. α and β diversity analyses were performed on OTU. Biom table of 16S rRNA gene sequencing data of fecal samples collected after the treatment. Richness and diversity **(A)** were calculated *via* Chao1 richness estimate (top panel) and Shannon index (bottom panel), respectively. *P*-values were determined *via* unpaired *t*-tests. Diversity between samples was determined *via* principal coordinate analysis (PCoA) with Bray-Curtis dissimilarity to analyze and compare the community structure between control and YBG groups **(B)**. Statistical significance was evaluated using PERMANOVA.

Our previous report (4) showed that β-glucan administration is effective in delaying the onset of T1D in NOD mice when the oral treatment was initiated at pre-diabetic adult ages by impacting the composition of gut microbiota. Here, we examined if initiation of YBG treatment at juvenile age, a period of immature/maturing gut microbiota, can shape the structure and function of microbiota. Two-week-old pre-weaning, juvenile female NOD mice were treated with YBG or saline for either 7 or 30 days, and the compositions of fecal bacteria were assessed immediately after treatment by 16S rDNA sequencing. 16S rDNA sequencing data was analyzed to determine the impact of YBG treatment on fecal microbiota alpha and beta diversity. Alpha diversity analyses (Figure 1A) revealed that the fecal microbial diversity (measured *via* Shannon index) and richness (measured *via* Chao1) were relatively lower in both 7- and 30-day YBG treated mice compared to respective control groups. However, this difference was statistically significant in mice that were treated for 30 days but not for 7 days. This result suggests, as observed in our previous studies (4, 5, 19), that YBG treatment at juvenile ages results in diminished diversity, perhaps due to selective enrichment, of microbial communities. Beta diversity analysis was performed *via* Principal Coordinate Analysis (PCoA) using Bray-Curtis index with PERMANOVA to evaluate community structure post-treatment samples. Figure 1B shows that significant differences in the fecal microbial community structures between control and YBG-treated mice were observed only in mice that were treated for 30 days (*p* = 0.022). These results indicate that prolonged YBG treatment at juvenile ages alters not only the composition of the gut microbiota but also the overall community diversity and structure.

### Compositional Changes in the Gut Microbiota in Mice That Received YBG Treatment at Juvenile Age

**Figure 2:**
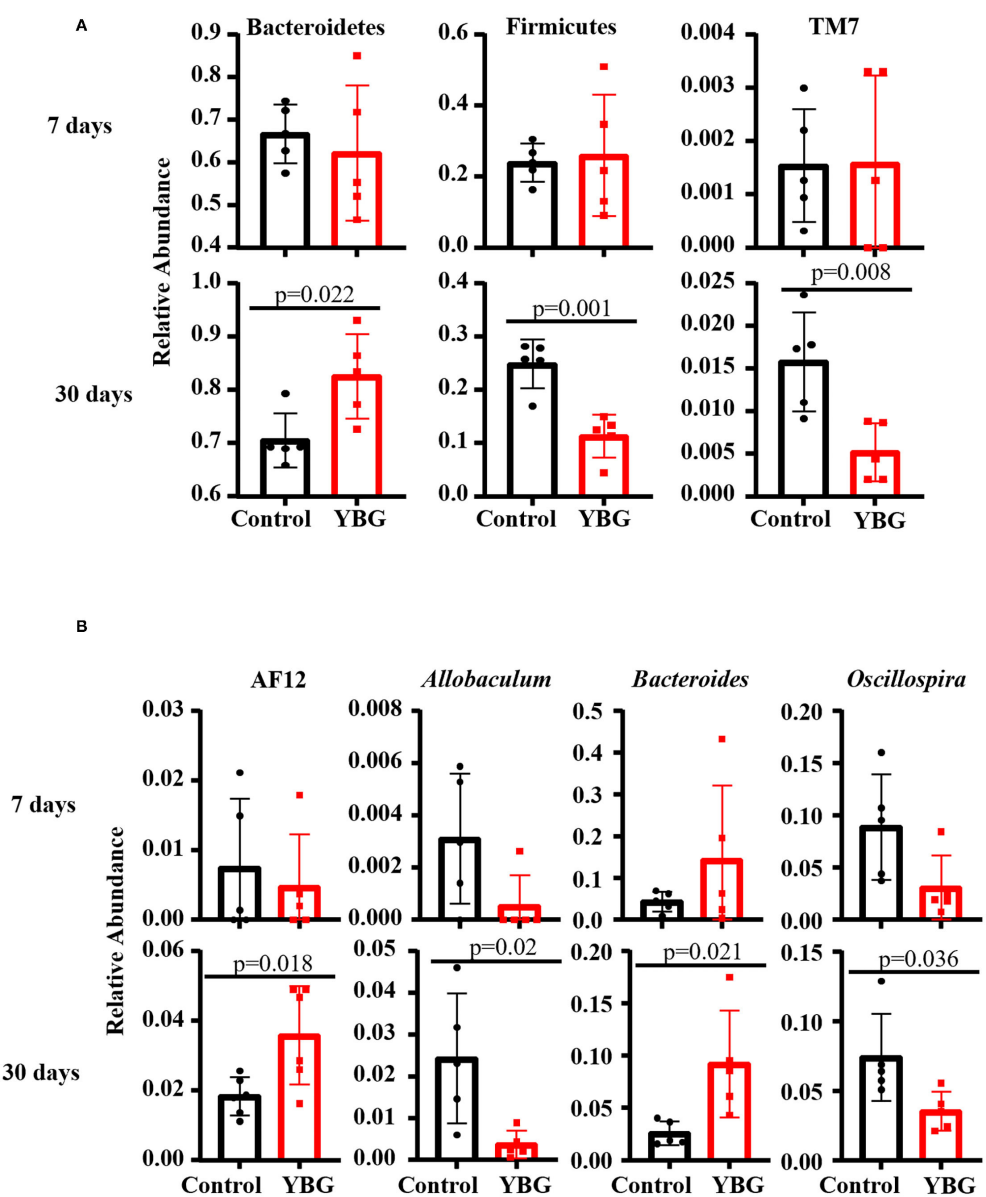
Differences in the fecal bacterial composition between control and YBG-treated NOD mice. Fecal samples of mice collected after 7 (5 mice/group) and 30 days (5 mice/group) of YBG treatment were subjected to 16S rRNA gene sequencing as described and analyzed employing Illumina Metagenomics application as described for Figure 1. The relative abundances of major microbial communities at phylum **(A)** and genus **(B)** levels with statistically significant differences are shown. *P*-values by unpaired *t*-test. Additional analyses are presented in Supplemental Figures 2, 3.

The 16S rDNA sequencing data, when GreenGenes database version 13.5 was employed as reference, revealed that fecal bacterial communities were dominated, at the phylum level, by Bacteroidetes, Firmicutes, Proteobacteria, and Verrucomicrobia in all groups (Supplemental Figure 1A). Gut communities of mice treated for 30 days were comprised 66 to 93% by Bacteroidetes, 4 to 28% by Firmicutes, 1 to 11% by Proteobacteria, and 0 to 7% by Verrucomicrobia. Between treatments, no differences in microbial communities at the phylum level were observed when the mice were treated with YBG only for 7 days. On the other hand, for mice treated for 30 days, YBG-treatment resulted in a significant increase in the abundance of Bacteroidetes (*p* = 0.022) and reduced levels of Firmicutes (*p* = 0.001) and TM7 (*p* = 0.008) compared to control mice (Figure 2A). At genus level, while there were no significant differences observed between 7-day YBG treated and respective control groups for mice, significantly higher abundance of AF12 (*p* = 0.018) and *Bacteroides* (*p* = 0.021) and significantly lower abundance *Allobaculum* (*p* = 0.02) and *Oscillospira* (*p* = 0.036) were observed in 30-day YBG treated mice compared to controls (Figure 2B, Supplemental Figure 1B). Additional differences in the microbial communities, mainly the communities depleted post-YBG treatment, at genus and species levels were identified when the data was analyzed employing Illumina Metagenomics application which used RefSeq RDP 16S v3 database (Supplemental Figure 2). Overall, these observations along with diversity and richness data indicate that prolonged treatment using YBG at juvenile age is necessary to exert major changes in the gut microbiota structure.

### YBG Treatment at Juvenile Age Causes a Shift in Gut Microbiota Function

**Figure 3:**
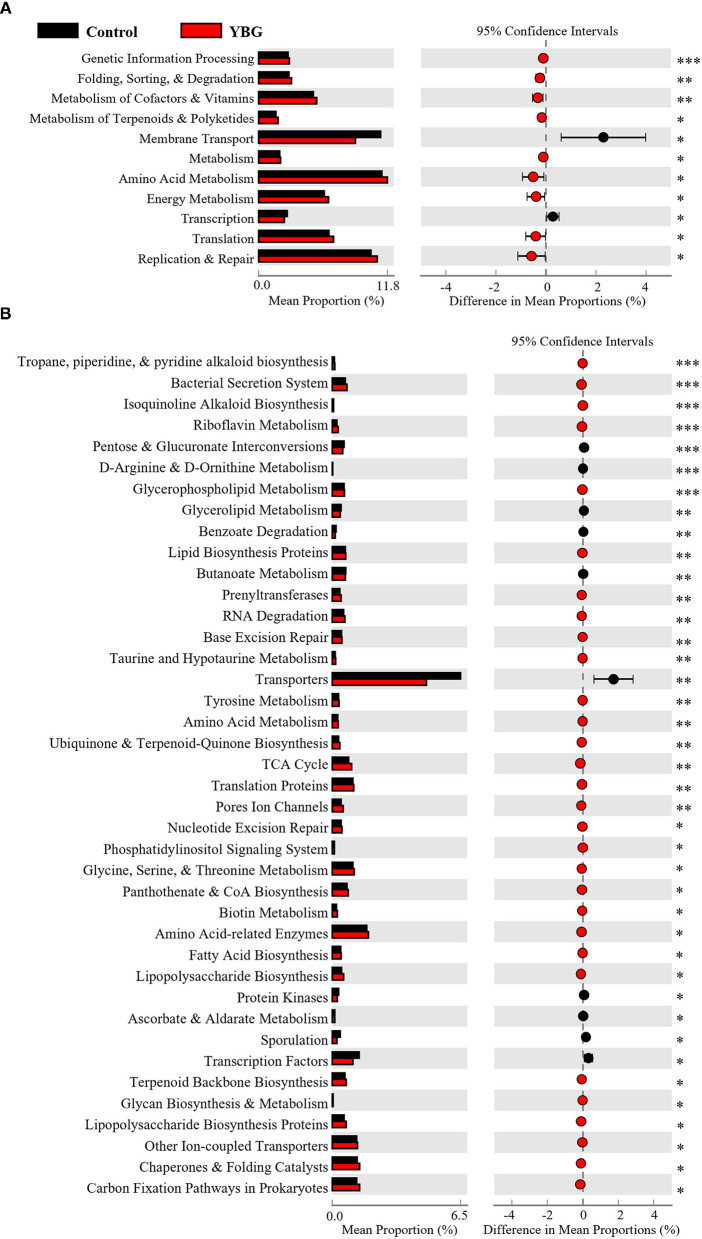
Predicted functions of fecal microbiota of NOD mice treated with YBG for 30 days. PICRUSt was used to predict level 2 **(A)** and level 3 **(B)** functional profiles, based on the 16S rRNA gene sequence, of the fecal microbiota of YBG treated and control groups of mice. STAMP was used to analyze and visualize significantly altered functions between control and YBG-treated mice after 30 consecutive days of treatment. No significant differences were observed after in mice that were treated only for 7 days (not shown). FDR corrected *p*-values are shown. *p < 0.05, **p < 0.01, ***p < 0.001.

To further assess the effects of YBG treatment at juvenile age on potential gut microbiota, PICRUSt was employed to predict the gut microbial functions from 16S rDNA sequences. This *in silico* analysis showed that short-term treatment with YBG for 7 days does not have an impact on the function of gut microbiota (not shown). However, the 2nd level of predictive functional hierarchy analysis showed overrepresentation of genetic information processing, energy metabolism, and the metabolism of cofactors and vitamins functions in fecal microbiota of mice treated with YBG for 30 days compared to controls (Figure 3A). On the other hand, membrane transport and transcription functions appear to be significantly underrepresented among the fecal microbiota of 30-day YBG-treated mice. The highest level (level 3) of predictive functional hierarchy analysis revealed that many microbiota functions, including glycan biosynthesis and metabolism, glycerophospholipid metabolism, and fatty acid biosynthesis were significantly overrepresented in the fecal microbiota of 30-day YBG-treated mice compared to controls (Figure 3B). These results, in addition to the upregulation of TCA cycle functions, indicate that prolonged YBG treated beginning at juvenile age causes a shift in the overall function of the gut microbiota toward glucose metabolism, as these suggest the increased breakdown of glucose through these means in YBG-treated mice. Further analysis of the predicted function *via* iVikodak identified *Prevotella* spp. as the likely primary contributors toward glycan degradation within the gut (Supplemental Figure 3). Further, *Bacteroides* spp. were more strongly linked to this function in YBG-treated mice, suggesting that YBG administration directly stimulates the growth of this taxon.

### Prolonged YBG Treatment at Juvenile Ages Alters Immune Phenotype of Colon

**Figure 4:**
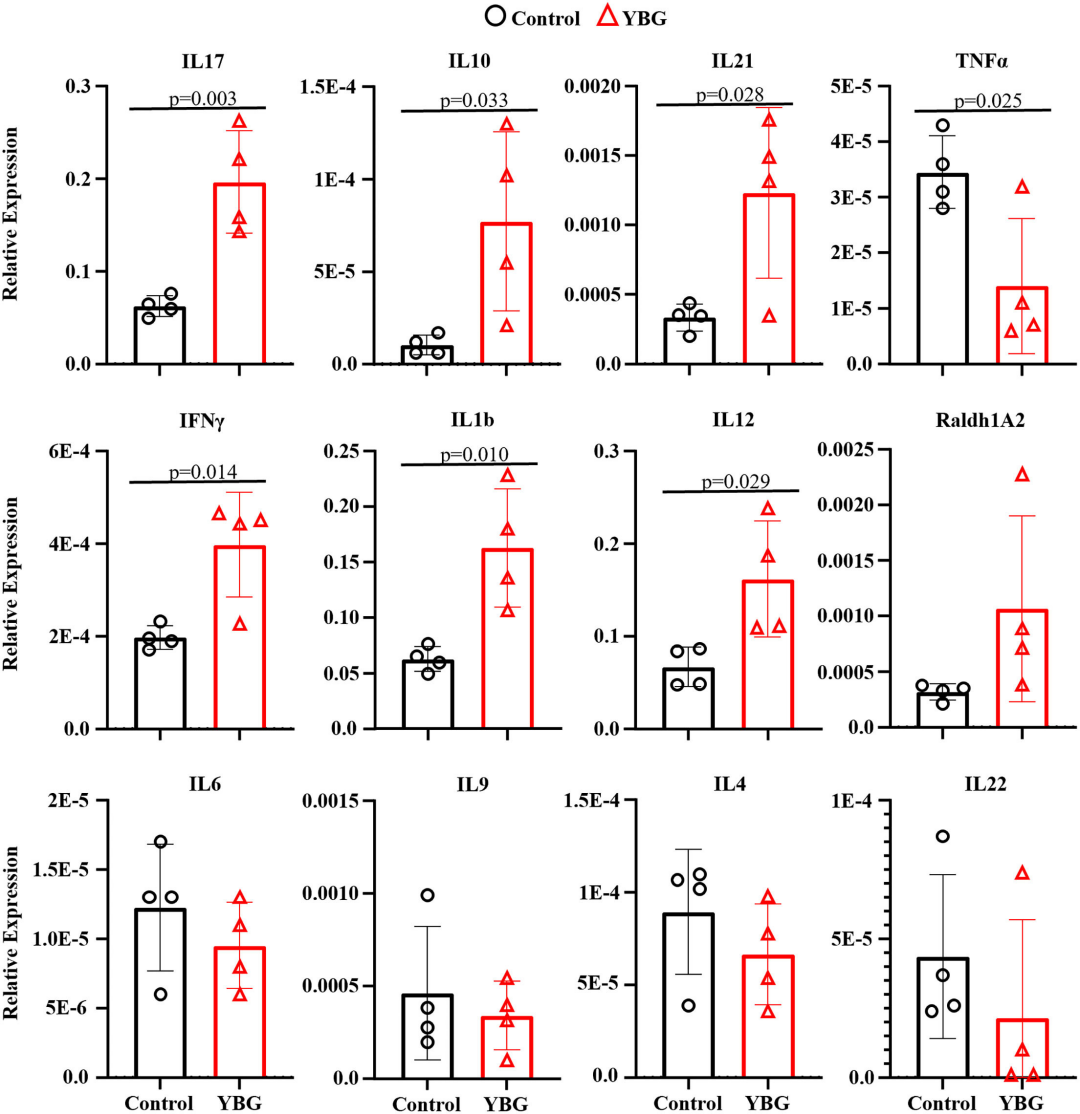
YBG treatment for 30 days alters the immune phenotype of large intestine of NOD mice. Two-week-old female NOD mice were given YBG or control saline *via* oral gavage for 7 (5 mice/group) or 30 days (4 mice/group) consecutive days and were euthanized 24 h after final treatment to harvest the intestine. cDNA was prepared from RNA extracted from the distal colon and RT-qPCR assay was performed to determine cytokine expression levels relative to β-actin expression. Assays were performed in triplicate for each sample, with *P*-values determined *via* Mann-Whitney test.

Since prolonged treatment of NOD mice starting at juvenile age with YBG caused changes in fecal microbiota, we examined if these changes impact the immune profile of gut mucosa by assessing the expression levels of key cytokines in the colon. Expression levels of various cytokines were not different in mice that received YBG treatment only for 7 days compared to their control counterparts (data not shown). In mice that received 30 days of YBG treatment, however, we observed higher expression levels of cytokines IL-10, IL-17, IFN-γ, IL-1b, IL-12, and IL-21 (Figure 4). A decrease in the expression of TNF-α was also observed. These results show that long-term treatment with YBG starting at juvenile age significantly alters the immune phenotype of distal gut.

### Prolonged YBG Treatment at Juvenile Ages Delays the Onset of Hyperglycemia in NOD Mice

**Figure 5:**
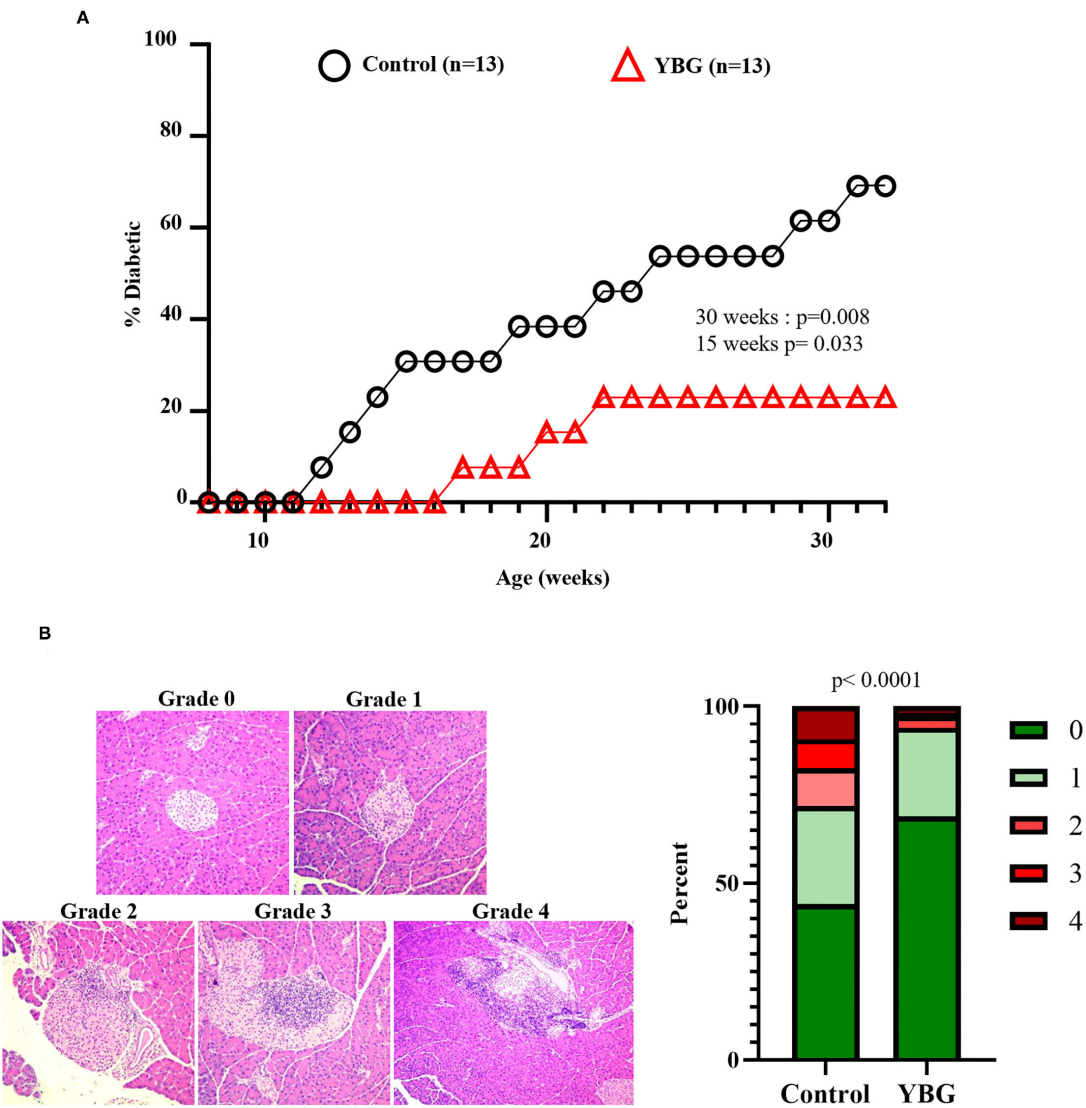
Impact of 30-day YBG treatment starting at juvenile age on eventual T1D disease onset and insulitis in NOD mice. Two-week-old female NOD mice were given saline or 100 μg YBG suspension for 30 consecutive days as described previously. Starting at 8 weeks of age, blood glucose levels of these treated mice were monitored weekly for up to 30 weeks of age to detect hyperglycemia onset and the overall T1D incidence **(A)**. Pancreatic tissues were collected from an additional cohort of control and YBG treated mice (4 mice/group) and the sections were subjected to H&E staining to assess the insulitis severity **(B)**. Representative images showing insulitis severity grades (left panel) and the percentage of islets with different insulitis grades (right panel) are shown. Statistical analysis was performed using a log-rank test **(A)** and Fisher's exact test **(B)**.

Our previous report (4) has shown that YBG treatment at pre-diabetic adult age is effective in delaying the onset of hyperglycemia. However, it is not known if YBG treatment starting at juvenile age can impact eventual disease outcomes in NOD mice. Since prolonged YBG treatment of starting at juvenile mice produced changes in the gut microbiota composition and function, and altered the immune phenotype of gut mucosa, we examined the effect of these treatments on the T1D disease outcomes. Supplemental Figure 4 shows that there is no difference in the timing of T1D onset in NOD mice that were treated with YBG only for 7 days compared to their control counterparts. However, mice treated with YBG for 30 days showed a significant delay in the onset of hyperglycemia compared to controls (Figure 5A). A significantly lower (*p* = 0.008) proportion of YBG treated mice were diabetic (28%) at 30 weeks of age compared to the control group (61%).

To assess the impact of prolonged YBG treatment starting at juvenile age on insulitis, cohorts of 30-day YBG or control treated mice were euthanized immediately after treatment (6 weeks of age) and pancreatic tissue sections were subjected to histopathological analysis after H&E staining. Figure 5B shows that islets of YBG treated mice had significantly less severe immune cell infiltration and damage compared to that of control mice (*p* < 0.0001). Approximately 6% of islets in mice treated with YBG showed an insulitis severity grade of 2 or more, while over 25% of islets in control mice showed this level of insulitis. These results indicate that treatment for a considerable duration is needed for preventing the onset of T1D even when the treatment is initiated at juvenile age. Nevertheless, these results show that YBG-induced modulation of autoimmunity is long lasting and persists even after the treatment is stopped long before the clinical disease onset stage. These results also suggest the possibility that continued oral consumption of YBG could help maintain YBG-shaped gut microbiota and immune environment, either directly or indirectly leading to further delayed disease onset.

## Discussion

While the impacts of β-glucan treatments on the gut microbiota and host immune system modulation have been investigated at the adult stage (4–6, 19, 20), how these prebiotic dietary complex polysaccharides modulate gut microbiota and immune function when the treatment is initiated at juvenile age is not known. The current study supports the prebiotic effects of YBG and other β-glucans even with treatment beginning in juvenile age, as seen with adult host models (4, 5, 19). Our data shows that prolonged treatment with YBG alters both the immune phenotype and gut microbiota function and improves disease outcomes in T1D-susceptible mice even when the treatment is initiated at juvenile age and stopped long before the disease onset age. Although 7-day YBG treatment starting at 2 weeks of age was insufficient, 30 days of treatment was sufficient to cause noticeable change in gut microbiota communities and gut immune phenotype and the overall disease outcome.

Oral administration of juvenile NOD mice with YBG altered abundance of some members of the gut microbiota, including Bacteroidetes and Firmicutes. Similar to adult mice (4, 5), prolonged treatment with YBG beginning at juvenile age significantly increases the relative abundance of Bacteroidetes and decreased the levels of Firmicutes in the gut. The decrease of TM7, a Gram-positive bacterial phylum that has been linked to bowel inflammation (34), with treatment is similar to that observed with probiotic treatment (35), indicating a potential indirect effect on the population of this phylum in the gut upon stimulation of natural probiotics with YBG supplementation. The increase in the genus *Bacteroides* and its link to glycan degradation with YBG treatment could be indicative of an increase in production of short-chain fatty acids (SCFA), which have immune system modulating properties (36), indicating a potential link between gut microbiota and the observed protection from autoimmunity. Commensal *Bacteroides* spp. are highly efficient at utilizing these complex dietary polysaccharides (37), so their increase here should be expected. AF12, an unculturable taxon, was also increased in YBG-treated mice, and has previously been linked to weight control and is also suggested to have a potential role in SCFA production (38). The decrease in richness and diversity within the gut microbiota after 30 days of β-glucan treatment starting at juvenile ages is similar to what has been observed previously in adult mice (4, 19). This could be indicative of the selective enrichment of specific members of the gut microbiota by YBG like prebiotics. PCoA analysis revealed that the community structures within the gut of control and YBG-treated mice were significantly different with prolonged treatment, providing further evidence of the influence of YBG on not only the composition but also the abundance of members within the gut microbiota.

Overrepresentation of functions related to glycan biosynthesis and fatty acid biosynthesis are characteristic of changes observed upon treatment with β-glucans in adult mice (4, 19). Increases in these functions suggest that YBG is being degraded in the colon and as shown in our previous report (4, 5), leading to the production of SCFA. SCFAs, which are produced by several commensal bacteria within the gut, have been linked to reductions in disease severity of several diseases through a shift away from a Th1-type response and toward Treg cell proliferation, reducing inflammation associated with autoimmunity (39, 40). The decrease in functions related to transporters and membrane transport with YBG treatment could indicate strengthening of the gut integrity, which is characteristic of the prebiotic effects linked to the reduction in gut inflammation seen with diabetes (41, 42).

Our previous report showed that the beneficial effects of YBG treatment on disease outcomes are limited to treatment initiated at pre-diabetic adult age but not at diabetic stage (4). Our current data shows that YBG treatment initiated at juvenile age for 30 days causes a significant reduction in islet infiltration/destruction by immune cells and delay in the onset of hyperglycemia in NOD mice irrespective of the fact that treatment was stopped long before the disease onset age. Since only 30-day treatment but not 7-day treatment had an impact on disease outcome, this suggests that prolonged treatment using this prebiotic agent through pre-diabetic ages could potentially prolong the protection from the clinical onset of disease further. These data, along with our previous report (4), supports the potential beneficial effects of YBG consumption as a dietary supplement at pre-clinical stages to prevent the disease in at-risk subjects. Analysis of the cytokine expression within the gut did show some evidence of a shift toward a Th17 type response with prolong YBG treatment, with increases in IL-10, IL-17, and IL-21 and a decrease in TNF-α. Previous reports have shown evidence of this shift away from a Th1-type response believed to trigger autoimmunity with T1D (4, 25). However, evidence showing increases in proinflammatory cytokines like IL-1b and IFN-γ reveal a need for further investigation into the changes brought on within the immune environment within the gut.

In conclusion, dietary supplementation of YBG beginning in infancy and at pre-clinical stages can alter the gut microbiota composition, diversity, and function, which either directly or indirectly enhances immune regulation and promotes protection from T1D. We herein provide evidence that such YBG treatment regimen is linked to delayed onset of hyperglycemia, as well as an altered immune phenotype that is potentially immune regulatory and Th17 biased. The change in microbiota and functioning within the gut when the YBG treatment is initiated at juvenile age is similar to what has been reported in adult mice, indicating that beginning treatment in infancy and continuing it may be a valid option for genetically pre-disposed individuals for establishing a stable host friendly microbiota. Importantly, studies involving the effect of treatment at early life in the absence of microbiota using germ-free mice as well as investigations of the direct role of β-glucan receptor, Dectin-1, at these ages are needed to further support the microbiota dependent mechanisms of protection from T1D achieved upon treatment with YBG.

## Data Availability Statement

The datasets presented in this study can be found in online repositories. The names of the repository/repositories and accession number(s) can be found at: NCBI SRA, PRJNA761366.

## Ethics Statement

The animal study was reviewed and approved by Institutional Animal Care Committee of Medical University of South Carolina.

## Author Contributions

HT designed experiments, researched and analyzed data, and wrote manuscript. CV designed experiments and reviewed and edited manuscript. All authors contributed to the article and approved the submitted version.

## Funding

This work was supported by National Institutes of Health (NIH) grants: R21AI133798 (NIAID), R21AI136339 (NIAID), R21AI133798-administrative supplement (ODS), and R21AI136339-02S1 and R01AI138511-02S2 Administrate supplements (ODS) to CV.

## Conflict of Interest

The authors declare that the research was conducted in the absence of any commercial or financial relationships that could be construed as a potential conflict of interest.

## Publisher's Note

All claims expressed in this article are solely those of the authors and do not necessarily represent those of their affiliated organizations, or those of the publisher, the editors and the reviewers. Any product that may be evaluated in this article, or claim that may be made by its manufacturer, is not guaranteed or endorsed by the publisher.

## Abbreviations

CDP: Complex Dietary Polysaccharide
IL: Interleukin
IFN: Interferon
NOD: Non-obese Diabetic
OTU: Operational Taxonomic Units
PICRUSt: Phylogenetic Investigation of Communities by Reconstruction of Unobserved States
PM: Paramylon
qPCR: quantitative Polymerase Chain Reaction
SCFA: Short-chain Fatty Acid
SPF: Specific Pathogen-free
STAMP: Statistical Analysis of Metagenomic Profiles
TLR: Toll-like Receptor
TNF: Tumor Necrosis Factor
T1D: Type 1 Diabetes
YBG: Yeast β-glucan.
