# Supplementary figures and images for: Impact of Prebiotic β-glucan Treatment at Juvenile Age on the Gut Microbiota Composition and the Eventual Type 1 Diabetes Onset in Non-obese Diabetic Mice
Source: Front Nutr. 2021 Nov 3;8:769341. doi: 10.3389/fnut.2021.769341 (PMC8595985; doi:10.3389/fnut.2021.769341)

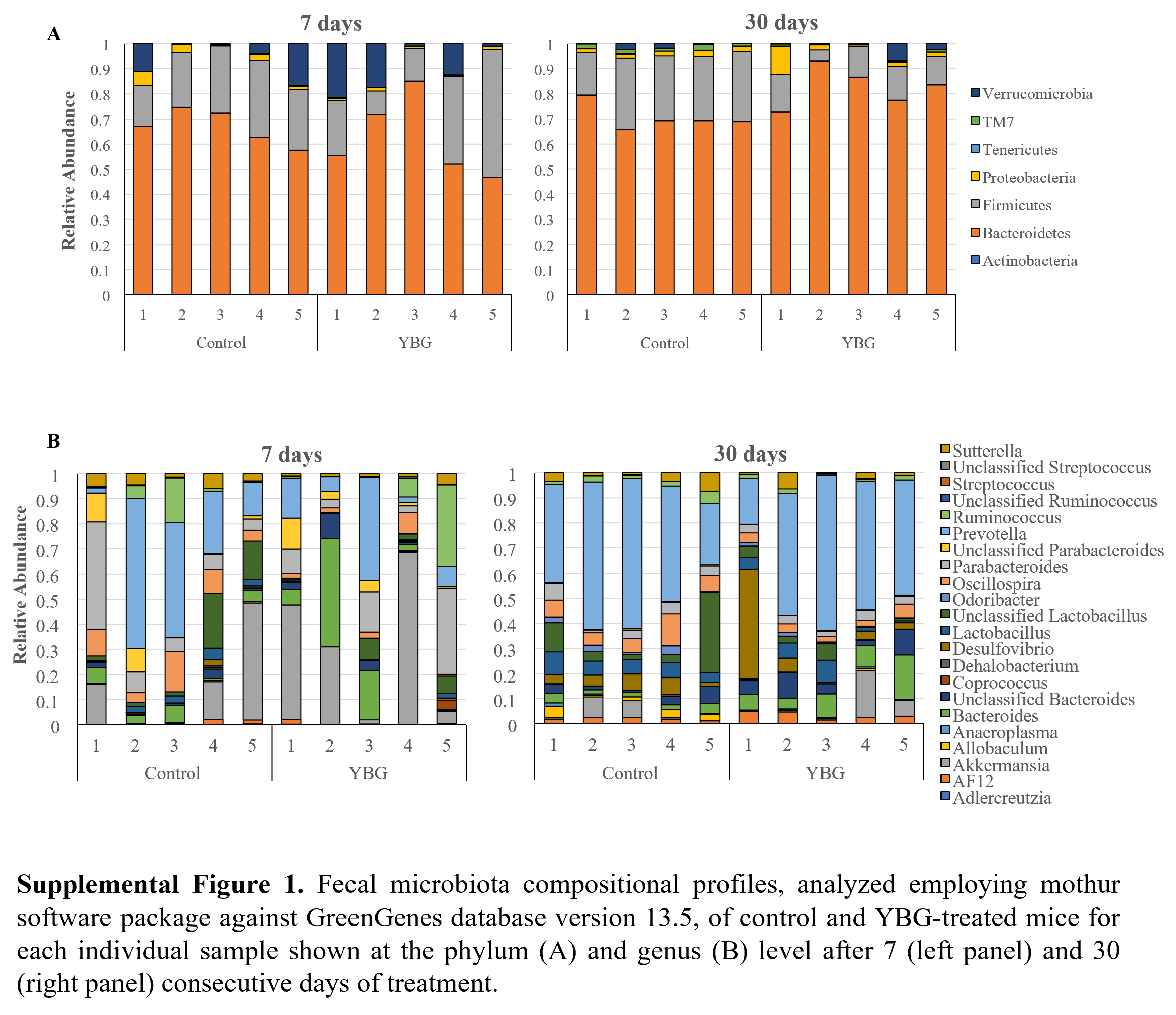

Supplement: Supplementary file 1 [file Image_1.TIF]

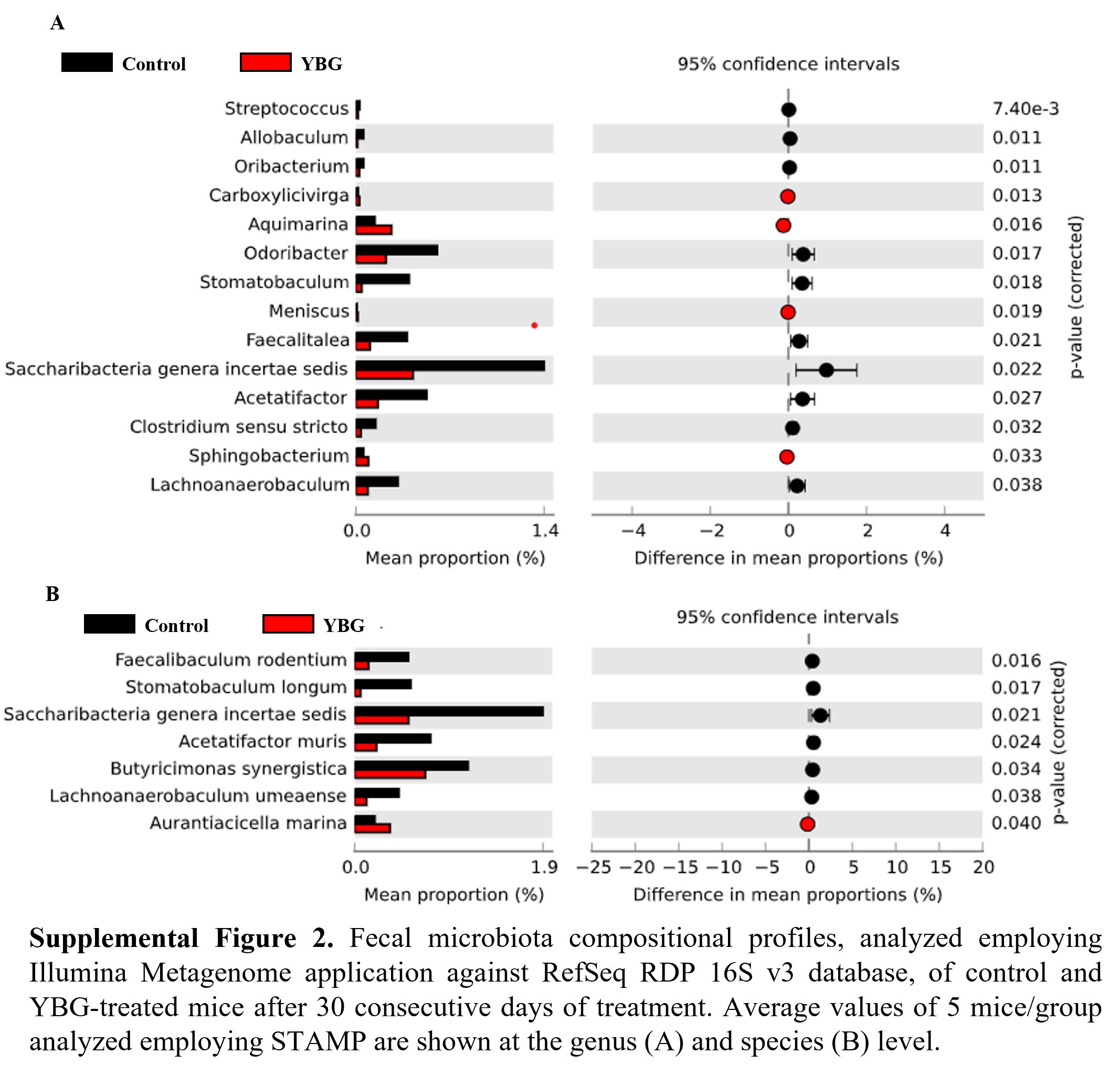

Supplement: Supplementary file 2 [file Image_2.TIF]

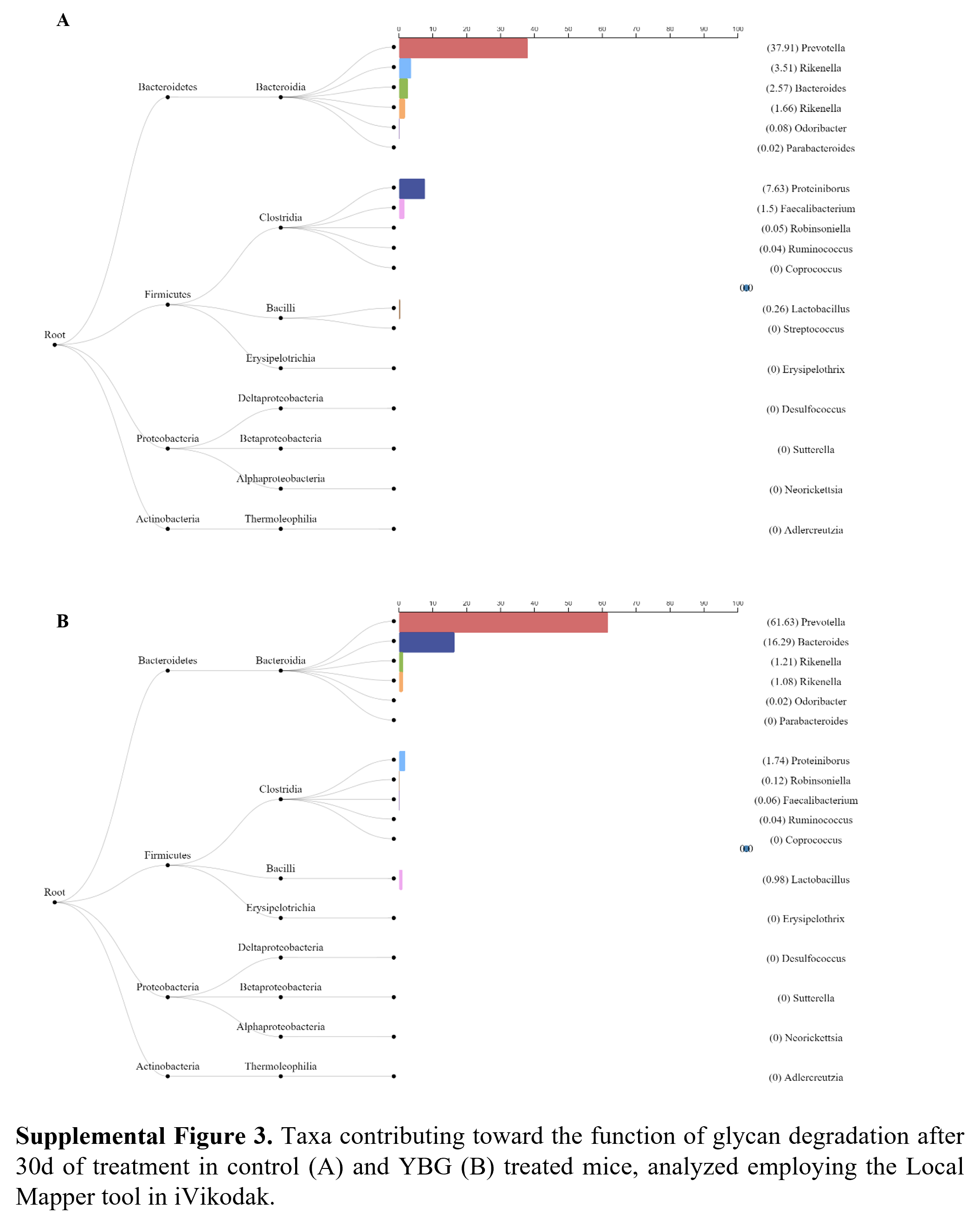

Supplement: Supplementary file 3 [file Image_3.TIF]

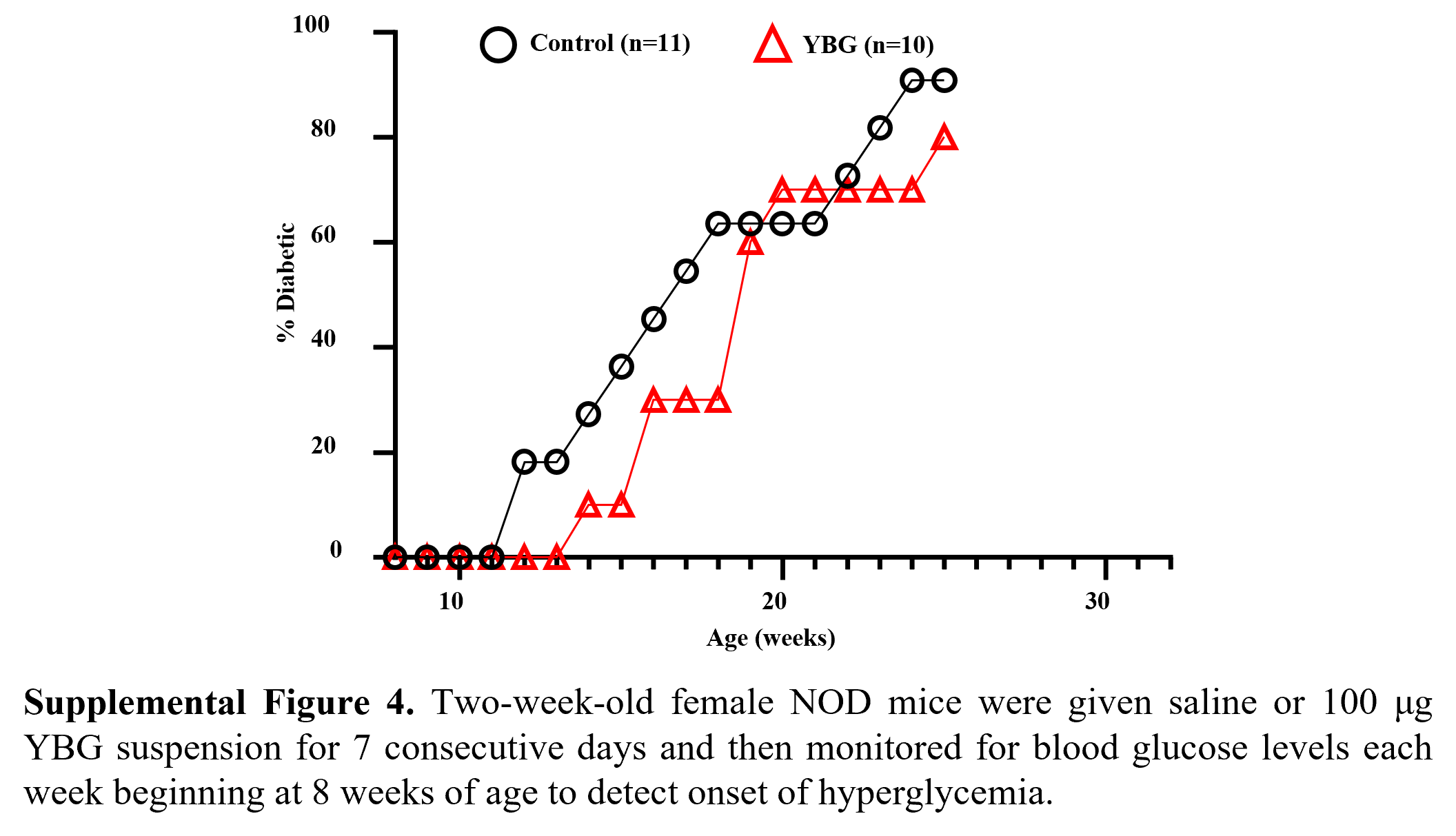

Supplement: Supplementary file 4 [file Image_4.TIF]

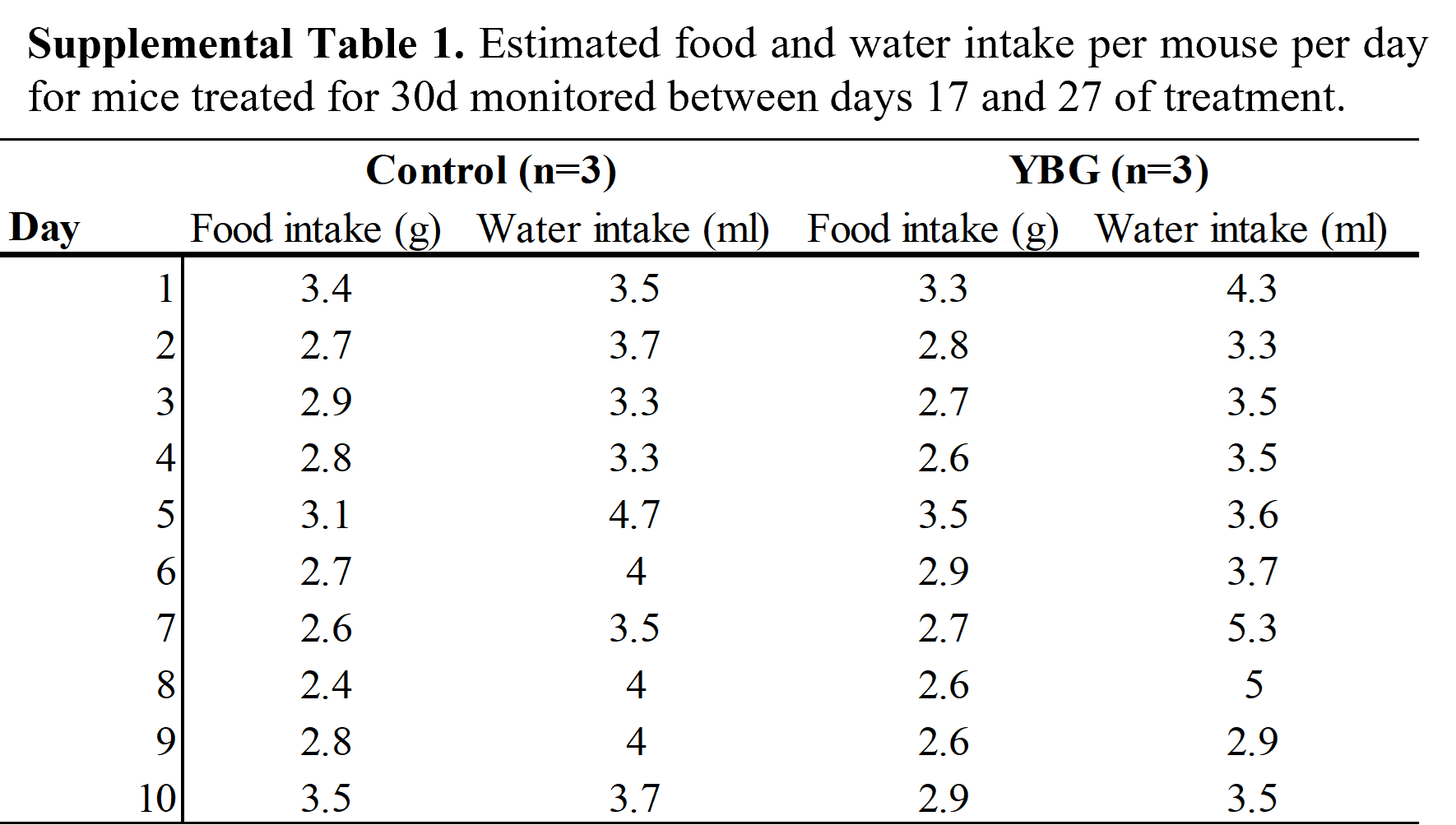

Supplement: Supplementary file 5 [file Image_5.TIF]
